# Ceftazidime–Avibactam in Multidrug-Resistant *Klebsiella* spp. Infections: Is Monotherapy as Effective as Combination Therapy?

**DOI:** 10.3390/antibiotics15020116

**Published:** 2026-01-25

**Authors:** Rukiyye Bulut, İbrahim Erayman, Bahar Kandemir, Pınar Belviranlı Keskin

**Affiliations:** 1 Department of Infectious Diseases and Clinical Microbiology, Necmettin Erbakan University Faculty of Medicine, Konya 42080, Türkiye; drerayman@yahoo.com (İ.E.); tekinbahar@hotmail.com (B.K.); 2 Department of Infectious Diseases and Clinical Microbiology, Konya City Hospital, Konya 42090, Türkiye; pinarbelviranli@gmail.com

**Keywords:** carbapenem-resistant *Klebsiella* spp., ceftazidime–avibactam, clinical success, mortality

## Abstract

**Background/Objectives**: Carbapenem-resistant *Klebsiella* spp. (CRK) causes healthcare-associated infections with high mortality. This study evaluated the clinical outcomes of ceftazidime–avibactam (CZA) therapy in CRK infections. **Methods**: Patients hospitalized in a tertiary care hospital in Türkiye between June 2021 and December 2022 with CRK-positive cultures, CZA susceptibility, and ≥72 h of CZA treatment were retrospectively analyzed. **Results**: Ninety-nine patients (61.6% male; mean age 63.7 ± 17.5 years) were included, 89.9% of whom were treated in the intensive care unit (ICU). Hypertension (29.3%), diabetes (28.3%), and malignancy (26.3%) were the most frequent comorbidities. The main infection types were bloodstream infection (56.6%) and ventilator-associated pneumonia (29.3%). CZA was used as monotherapy in 49.5%, and in combination in 50.5% of cases. The mean treatment duration was 13.2 ± 6.3 days. Clinical improvement occurred at 3.4 ± 1.2 days and microbiological eradication at 4.7 ± 2.1 days. Treatment success was achieved in 76.8% of patients, while 30- and 90-day mortality rates were 48.5% and 72.7%, respectively. Only treatment duration significantly affected clinical outcome (*p* < 0.001). **Conclusions**: CZA demonstrates favorable outcomes in CRK infections, with no significant difference between monotherapy and combination therapy. These findings support the use of CZA as an effective treatment option for severe CRK infections in real-world clinical settings and may help guide antimicrobial stewardship strategies in high-risk hospitalized patients.

## 1. Introduction

Resistance to antimicrobial agents in multidrug-resistant (MDR) Gram-negative bacteria is a global emergency. For many years, carbapenems have been considered as the most effective option for the treatment of infections caused by MDR Gram-negative bacilli, including species such as *Pseudomonas aeruginosa* and extended-spectrum beta-lactamase (ESBL)-producing *Enterobacteriaceae*. The recent emergence and spread of new types of β-lactamases capable of hydrolyzing carbapenems, including *Klebsiella pneumoniae* carbapenemases (KPCs) or various metallo-β-lactamases (MBLs), poses a challenge to the treatment of MDR bacteria [1,2,3]. Carbapenem-resistant *Klebsiella* spp. (CRK) infections increase mortality rates, prolong hospital stays, and increase healthcare costs [4]. According to the 2024 National Healthcare-Associated Infections Surveillance Report in Turkiye, carbapenem resistance in *Klebsiella* spp. is at a serious level, with 72.67%, at university hospitals [5]. Ceftazidime–avibactam (CZA) is a novel antimicrobial agent that combines an antipseudomonal cephalosporin with a beta-lactamase inhibitor. In vitro studies demonstrate that avibactam has antimicrobial activity against ESBL, AmpC, KPC, and oxacillinase-48 (OXA-48)-producing *Enterobacteriaceae* and MDR *P. aeruginosa* isolates [1,2,3,6]. Animal studies verify that CZA is effective against ceftazidime-resistant Gram-negative bacteremia, meningitis, pyelonephritis, and pneumonia. Its safety and tolerability in clinical trials have been excellent, and serious drug-related adverse events have been rare [2]. Real-life studies in the literature also provide evidence for the successful use of CZA in the treatment of carbapenem-resistant *Enterobacteriaceae* (CRE) and MDR Gram-negative bacterial infections [3]. The IDSA guideline recommends reserving CZA for the treatment of infections caused by carbapenem-resistant microorganisms [7]. CZA was approved in our country in 2018 for use in combination with metronidazole in complicated intra-abdominal infections in adult patients, for the treatment of complicated urinary tract infections including pyelonephritis, nosocomial pneumonia including ventilator-associated pneumonia (VAP), and infections caused by aerobic Gram-negative microorganisms with limited treatment options, and it was reimbursed by the Social Security Institution of the Republic of Turkiye in 2021 and became more widely used in hospitals [8].

This study aims to contribute to real-world data in Turkiye by investigating the efficacy and safety of CZA in real-life clinical practice in adult patients with documented CRK infection receiving inpatient treatment at a tertiary hospital.

## 2. Results

A total of 99 patients were included in the study: 61 (61.6%) males and 38 (38.4%) females. All patients were between 18 and 89 years of age, with a mean age of 63.7 ± 17.5.

Eighty-nine (89.9%) of the patients were admitted to the ICU [Reanimation ICU (25.5%), Internal Medicine ICU (23.2%), Chest Diseases ICU (18.2%), Neurosurgery ICU (14.1%), Neurology ICU (6%), General Surgery ICU (2%), and Cardiology ICU (1%)]. Eighty-three (83.8%) of the patients had a comorbidity in their medical history (Table 1). The diagnoses at presentation and admission to the hospital, in order of frequency, were malignancy and malignancy-related complications in 24 patients (24.2%), cerebrovascular accident in 16 patients (16.2%), COVID-19 in 12 patients (12.1%), trauma in 12 patients (12.1%), pneumonia in 11 patients (11.1%), and other causes in 24 patients (24.2%). The most common infectious diagnoses were bloodstream infection (BSI) (56.6%) and ventilator-associated pneumonia (29.3%) (Table 1).

When the patients’ laboratory parameters were evaluated, the mean CRP on the day of infection diagnosis was 169.9 ± 88.97, and the median CRP value at the end of treatment was 85. A significant difference was found between the CRP values at diagnosis and after treatment (*p* < 0.001). The median procalcitonin value on the day of infection diagnosis was 4.4, and the median procalcitonin value at the end of treatment was 1.35. A significant difference was found between the procalcitonin values at diagnosis and after treatment (*p* = 0.006) (Table 2).

The most common culture samples yielding CRK growth were blood (50.5%) and bronchoalveolar lavage fluid (30.3%).

Forty-nine (49.5%) patients received CZA as monotherapy, and 50 (50.5%) received it in combination with another antibiotic. The majority of patients (70.7%) had received other antimicrobials before or in combination with CZA. Patient treatment characteristics and outcomes are presented in Table 3.

The median dose of CZA administered to patients was 2.5 g (0.94–2.5 g) administered three times daily, and the mean treatment duration was 13.2 ± 6.3 days (3–28). Patients with renal impairment (*n* = 30) received their CZA dose according to their estimated glomerular filtration rate (eGFR).

Treatment success was achieved in 76.8% of patients at the end of therapy. Among the 76 patients who were considered a treatment success for the CRK episode, 26 patients died within the 30-day follow-up period due to causes unrelated to the primary CRK infection (such as progression of underlying malignancy, respiratory failure, or secondary superinfections). Consequently, while the specific CRK-attributable mortality was 17.2%, the all-cause 30-day and 90-day mortality rates were 48.5% and 72.7%, respectively. Patient mortality outcomes are shown in Table 4.

When treatment success and failure at the end of therapy were considered as the primary endpoint, no significant differences were observed with respect to gender, use of CZA monotherapy versus combination therapy, infection type, or admission diagnosis. Only duration of treatment was found to be a significant difference in the primary endpoint (*p* < 0.001) (Table 5).

## 3. Discussion

Prior to the introduction of CZA, combinations of multiple antibiotics, such as colistin, tigecycline, meropenem, and gentamicin, or in selected cases, dual carbapenem therapy, were widely considered superior to single-drug regimens, particularly in the treatment of severe CRE infections. However, many of these molecules have significant limitations related to efficacy data, unfavorable pharmacokinetic/pharmacodynamic profiles, and toxicity [2,9]. CZA is the last major weapon for treating infections caused by carbapenem-resistant bacteria [2]. The Infectious Diseases Society of America (IDSA) guideline recommends CZA for the treatment of infections caused by carbapenem-resistant organisms [7].

This study presents the data on the real-life hospital use of CZA in CRE-related infections (type of infection treated, route of administration), clinical efficacy (clinical response and mortality), and safety. In our study, 89.9% of the patients were in the ICU, the mean age was 63.7 ± 17.8 years, and 61.6% were male. Studies on this topic in adults show that the majority of patients were male, and the mean age was similar to our study [3,10,11,12].

In this study, 83.8% of the patients had comorbidities, the most common of which were hypertension (29.3%), diabetes mellitus (DM) (28.3%), solid organ malignancy (26.3%), active chemotherapy (19.2%), coronary artery disease (17.2%), hematological malignancy (14.1%), and hemodialytic chronic kidney disease (CKD) (9%). In the study by Soriano et al., immunodeficiency was determined in 47.1% of the patients and moderate/severe CKD in 21.9% of the patients due to solid organ or hematological malignancy, bone marrow or solid organ transplantation, or acquired immunodeficiency syndrome (AIDS) [3]. In a multicenter study conducted in France with 257 patients, the most common comorbidities were DM (30%), CKD (25.7%), and immunosuppression (31.1%) [13].

With regard to clinical indications, the most common indications for CZA use were BSI (56.6%), VAP (29.3%), and intra-abdominal infections (6.1%). In a multicenter retrospective cohort study conducted in the United States (US) by Jorgensen et al., the major indications for CZA use were reported as respiratory (37.4%), urinary (19.7%), intra-abdominal (18.7%), skin and soft tissue (8.9%), and osteoarticular (6.9%) infections [1]. In the literature, CZA is most commonly used for pneumonia, bacteremia, skin and soft tissue infections, urinary tract infections, and intra-abdominal infections [3,10,11,12,14].

In the study cohort, 49.5% of patients received CZA as monotherapy, while 50.5% received it in combination with another antibiotic. When factors affecting post-therapy response were analyzed, no significant difference was observed between CZA monotherapy and combination therapy. Similarly, Yu et al. reported that CZA monotherapy and its combination with other agents did not affect 14-day clinical response or 90-day survival [15]. Balandin et al. also found no significant difference in clinical response between monotherapy and combination therapy [14]. In contrast, Yang et al. reported that CZA monotherapy was associated with better outcomes [12]. Another retrospective study also found no significant difference in mortality between patients treated with CZA alone and those treated with combination regimens [9]. According to the IDSA 2024 guideline, CZA monotherapy is recommended for infections caused by KPC/OXA-48-producing CRE and difficult-to-treat *Pseudomonas* spp. [7]. Taken together, these findings suggest that CZA monotherapy provides comparable efficacy to combination therapy in most clinical settings, particularly when the pathogen is susceptible. However, the choice between monotherapy and combination therapy should still consider the local prevalence of NDM producers and patient-specific clinical factors.

In this analysis, the mean duration of CZA treatment was 13.2 ± 6.3 days, and successful treatment responses were achieved in 76.8% of patients. In the study by Soriano et al., the median duration of treatment was 9 (range, 7–14) days, and treatment success was achieved in 77.3% of patients [3]. In a study by Balandin et al. on patients in the ICU, the median duration of treatment was 10 days, and a 73.5% clinical improvement was observed [14]. In another retrospective study, the mean duration of CZA treatment was 6.92 ± 4.1 days, and clinical success was achieved in 76.3% of patients at the end of treatment [16].

Regarding clinical outcomes, mortality due to CRK infection during CZA treatment was observed in 22 patients (17.2%), and the 30-day mortality rate was 48.5%. In a multicenter prospective study conducted in Greece, 28-day mortality was significantly lower in patients receiving CZA monotherapy for pathogen-specific treatment (18.3%) than in patients receiving antibiotics other than CZA (40.8%) [17]. A meta-analysis of 11 real-world studies (396 patients total, 194 of whom received CZA monotherapy) reported a significantly lower mortality rate in patients treated with CZA compared to colistin-based regimens. No significant differences were found between monotherapy (mortality 30.9%) and combination therapy (mortality 38.1%) in terms of microbiological cure and mortality rates [18]. In most publications containing real-world data, clinical success rates with CZA treatment range from 45 to 100%, depending on the type of infection, pathogen, and patient characteristics, and 30-day mortality rates have been reported as 0–63% [11]. Increasing rates of NDM-positive CRE over the years are one of the reasons for the failure of CZA treatment [19].

With respect to longer-term outcomes, 5 patients (5.1%) developed reinfection or relapse during the 90-day follow-up period, while the overall 90-day all-cause mortality rate was 72.7%. The reinfection rate was notably lower than that reported by Calvo-García et al. (36.5%) [10], which may be attributed to differences in patient selection, infection source, or duration of antimicrobial therapy. However, the 90-day mortality in our cohort was substantially higher compared with that reported by Yu et al. (39.5%) [15]. This discrepancy could be explained by the high proportion of critically ill patients and the possible predominance of NDM-producing *Enterobacterales*, which are associated with limited therapeutic options and poorer clinical outcomes.

In our study, no significant differences were observed in treatment success according to age, gender, infection site, or admission diagnosis. Although treatment duration was identified as a parameter significantly associated with clinical success (*p* < 0.001), this finding should be interpreted with caution due to the potential for survivorship bias (reverse causality). The significantly shorter mean duration observed in the failure group (6.9 ± 5.8 days) largely reflects early mortality among critically ill patients, which inherently limited the duration of therapy. Therefore, the association between longer treatment duration and treatment success is more likely attributable to survival bias, as patients who survived long enough were able to complete the treatment course, rather than to a causal effect of prolonged treatment. In contrast, Soriano et al. reported that older age, complicated intra-abdominal infections, and nosocomial pneumonia/VAP indications, as well as concomitant colistin use, were independent predictors of reduced clinical success, while gender, bacteremia, immunocompromised status, and renal dose adjustment were not significantly associated with outcomes [3]. Similarly, another retrospective study identified lower respiratory tract infection as a factor associated with increased mortality [9]. The differences between studies may reflect variations in patient populations, infection severity, resistance mechanisms, and local treatment practices. In particular, in our cohort, the impact of prolonged treatment duration on success may also be related to the predominance of multidrug-resistant pathogens and the need for extended therapy in critically ill patients.

CZA is associated with a low rate of side effects [9]. In the study by Soriano et al., 6 of 569 patients experienced adverse events, including altered consciousness, neurotoxicity, cholestasis, hepatocellular injury, multiple organ dysfunction syndrome, Clostridium difficile colitis, and Stevens-Johnson syndrome [3]. In the study by Calvo-Garcı et al., treatment was discontinued due to adverse events in 4.8% of patients [10]. In a multicenter retrospective cohort study conducted in the USA, 8.4% of patients experienced CZA-related adverse events (10 acute kidney injury, 3 Clostridioides difficile infections, 2 rashes, and 1 gastrointestinal intolerance and neutropenia) [1]. In a meta-analysis by Shields et al. (24 real-world studies, mostly including patients with serious infections), CZA-related adverse events were reported as mild diarrhea, encephalopathy, mild increases in blood urea and creatinine, increased transaminases, increased alkaline phosphatase (ALP), gamma-glutamyl transferase (GGT), and total bilirubin, and thrombocytosis [20]. In our study, only one patient experienced diarrhea as a side effect. This finding supports the favorable safety profile of CZA observed in clinical trials and real-world studies. The low rate of adverse events in our study may also reflect careful patient monitoring and the limited duration of therapy in some cases.

The limitations of our study are that it is a single-center, observational, retrospective study and the sample size is relatively small. However, as a university hospital, most of our sample consisted of critically ill patients in the ICU who were regularly monitored by the infection control team. Since the study population consisted solely of documented CRK-related infections, and most of these were BSIs, our results will provide guidance for difficult-to-manage CRK infections.

Despite these limitations, future studies should focus on prospective, multicenter designs to further define the optimal role of CZA in the treatment of CRK infections. Comparative evaluations of monotherapy versus combination therapy, as well as long-term follow-up to assess the emergence of resistance, would provide valuable insights. In addition, investigations addressing species and subspecies-specific responses among *Klebsiella* isolates and the integration of CZA use within antimicrobial stewardship programs may help optimize clinical outcomes while preserving antimicrobial efficacy.

Due to the increasing rates of antimicrobial resistance, CRK infections pose a serious public health threat. Combination antibiotic therapies used in the treatment of these infections may lead to increased toxicity and higher treatment costs. Our findings suggest that CZA is an effective and safe therapeutic option for the management of severe CRK-related infections, including selected cases where it can be used as monotherapy. However, CZA should not be considered a universal replacement for other treatment strategies, and its use should be carefully guided to minimize the risk of overuse and the potential development of resistance. Integration of CZA into antimicrobial stewardship programs is therefore essential.

## 4. Materials and Methods

### 4.1. Study Design and Patient Selection

This is a retrospective, observational study. Patients aged ≥18 years, who received CZA treatment as an inpatient at a tertiary care training and research hospital with 1.350 beds in Türkiye between 1 June 2021, and 31 December 2022, and who were found to have grown carbapenem-resistant *Klebsiella* species (CRK) in any culture sample, who were found susceptible to CZA based on an antibiogram, and received at least 72 h of CZA treatment were included in the study. Patients for whom CRK growth was considered colonization, those who had empirically initiated CZA, and those with no culture growth were excluded from the study. Data regarding the patients included in the study: age, gender, comorbidities, presence of immunosuppression, admission diagnosis, symptoms, date of infection diagnosis, type of infection, use of additional antimicrobial therapy, type of specimen that yielded growth, starting dose of CZA therapy, mono/combination therapy, day of clinical recovery, day of microbiological recovery, duration of treatment, side effects, post-therapy response, cause of mortality (infectious/non-infectious), C-reactive protein (CRP) and procalcitonin levels at the time of infection diagnosis, CRP and procalcitonin levels at the end of CZA therapy, 30-day and 90-day mortality, and the data of relapse/reinfection status were retrospectively reviewed from patient files and the hospital information management system and recorded by an infectious disease specialist.

Infection diagnoses were made according to Centers for Disease Control and Prevention (CDC) criteria [21]. Only one CRK isolate from each patient was evaluated. CZA was used at a standard dose (2.5 g administered every 8 h, 2-h infusion), with renal dose adjustments made when required according to the package insert. All patients enrolled in the study were followed for 90 days to record their survival. Ethical approval for this study was obtained from the Necmettin Erbakan University Ethics Committee for Non-Drug and Non-Medical Device Research (on 19 July 2024, with decision number 2024/5112).

### 4.2. Treatment and Outcome Definitions

Successful post-therapy response was defined as improvement in clinical signs and symptoms with CZA treatment and discontinuation of antibacterial therapy. It is important to note that this definition strictly refers to the resolution of the specific CRK infection episode. Given the high severity of illness in the study cohort, patients who achieved clinical resolution of the CRK infection but subsequently died during the follow-up period due to underlying comorbidities (e.g., malignancy, advanced organ failure) or secondary non-CRK causes were classified as ‘treatment success’ for the primary endpoint, distinct from all-cause mortality. Unsuccessful post-therapy response included persistence of signs and symptoms of infection, death, or recurrence of infection. Combination therapy was defined as the use of a second antibiotic with in vitro activity against Gram-negative bacteria (specifically colistin, tigecycline, aminoglycosides, carbapenems, fosfomycin, or quinolones) in combination with CZA for the treatment of CRK. Patients receiving concomitant antimicrobials solely for Gram-positive or fungal coverage (e.g., vancomycin, teicoplanin, caspofungin) without an additional Gram-negative active agent were classified as receiving CZA monotherapy for the purpose of this analysis. The primary endpoints of the study were to describe the post-therapy response, clinical improvement, and microbiological eradication after initiation of CZA treatment. CZA-related adverse events were also recorded. Secondary endpoints included the indication for CZA use, dose (dosage frequency, duration, concomitant antimicrobials), the incidence of relapse/reinfection (due to CRK) during hospital stay, and 30- and 90-day mortality. Patients with polymicrobial cultures (isolating CRK alongside other pathogens) were included in the analysis. In such cases, appropriate concomitant antimicrobial therapy targeting the non-CRK pathogens (e.g., anti-Gram-positive or antifungal agents) was administered in addition to CZA. For these patients, treatment success was defined as the clinical resolution of the infection under a regimen effective against all isolated pathogens. It was assumed that clinical improvement required the effective eradication of the CRK component by CZA, as untreated CRK would likely result in persistent infection or failure.

### 4.3. Microbiological Evaluation

The VITEK^®^ 2 automated system (bioMérieux, Marcy-l′Étoile, France) was used for initial identification of microorganisms and antimicrobial susceptibility testing. All isolates were identified as *Klebsiella* species, and species- and subspecies-level identification was further confirmed using matrix-assisted laser desorption/ionization time-of-flight mass spectrometry (MALDI-TOF MS; bioMérieux, Marcy-l′Étoile, France). Briefly, bacterial colonies obtained from overnight cultures were applied to a stainless-steel target plate, overlaid with a matrix solution, and analyzed via MALDI-TOF MS. Identification was achieved by comparing the generated mass spectra with the manufacturer’s reference database, allowing discrimination at the species and subspecies levels.

All isolates were tested for susceptibility to β-lactam, aminoglycoside, and quinolone antibiotics. Minimum inhibitory concentrations (MICs) were interpreted according to the European Committee on Antimicrobial Susceptibility Testing (EUCAST) guidelines [22]. Carbapenem resistance was defined as resistance to ertapenem at an MIC > 0.5 mg/L and to imipenem or meropenem at an MIC > 8 mg/L.

### 4.4. Statistical Analyses

Statistical analyses were performed using IBM SPSS Statistics for Windows, Version 26.0 (IBM Corp., Armonk, NY, USA, 2019). Descriptive statistics were used to summarize demographic and clinical data. Normality of distribution was assessed using the Kolmogorov–Smirnov and Shapiro–Wilk tests. Normally distributed variables were expressed as mean ± standard deviation (SD), and non-normally distributed variables were expressed as median and interquartile range (Q1–Q3). Categorical data were presented as number (*n*) and percentage (%). Patients were divided into two groups: successful and unsuccessful based on their post-therapy response. To compare demographic, clinical, and laboratory parameters between the groups, the following statistical analyses were employed: Student’s *t*-test was used for normally distributed continuous variables, the Mann–Whitney U test for non-normally distributed continuous variables, and the chi-square test for categorical variables. Additionally, the relationship between pre- and post-treatment procalcitonin and CRP levels was assessed using the Wilcoxon signed-rank test.

## Figures and Tables

**Table 1:** Demographic and clinical characteristics of the patients.

	Characteristics (Unit)	*n* (%)
Gender	Male	61 (61.6)
	Female	38 (38.4)
Age (years)	Mean ± SD	63.7 ± 17.5
Clinic	Intensive Care Unit	89 (89.9)
	General Wards	10 (10.1)
Comorbidities *		83 (83.8)
	Hypertension	29 (29.3)
	Diabetes Mellitus	28 (28.3)
	Solid Organ Malignancy	26 (26.3)
	Receiving Chemotherapy	19 (19.2)
	Coronary Artery Disease	17 (17.2)
	Hematological Malignancy	14 (14.1)
	CKD (Requiring Dialysis)	9 (9.1)
	COPD	7 (7.1)
	Previous CVE	4 (4)
	Rheumatological Disease	4 (4)
	Liver Cirrhosis	3 (3)
	Immunodeficiency	2 (2)
	Auto-HSCT	2 (2)
	SOT	2 (2)
	Primary Immunodeficiency	2 (2)
	Alzheimer’s disease	1 (1)
Infectious Diagnoses **		
	Bloodstream Infection ***	56 (56.6)
	VAP	29 (29.3)
	Intra-abdominal Infection	6 (6.1)
	Pneumonia	4 (4)
	SSI	4 (4)
	Urinary Tract Infection	4 (4)
	Decubitus Infection	1 (1)

* Patients with multiple comorbidities are included. ** Patients diagnosed with multiple infections simultaneously are included. *** Catheter-associated bloodstream infections are also included. SSI: Surgical site infection, CKD: Chronic kidney disease, COPD: Chronic obstructive pulmonary disease, *n*: number, Auto-HSCT: Autologous hematopoietic stem cell transplantation, SOT: Solid organ transplantation, CVE: Cerebrovascular event, VAP: Ventilator-associated pneumonia.

**Table 2:** Laboratory parameters of the patients.

*n* = 99	Mean ± SD	Median (Q1–Q3)	z	*p*
CRP at diagnosis	169.9 ± 88.97		−4.660	<0.001
CRP at the end of treatment	108.16 ± 92.98	85 (31–157)		
Procalcitonin at diagnosis	14 ± 41.61	4.4 (0.8–10.5)	−2.739	0.006
Procalcitonin after treatment	12.41 ± 42.59	1.35 (0.38–3.93)		

CRP: C-Reactive protein; z: Test statistic of the Wilcoxon signed-rank test; *p* values were calculated accordingly.

**Table 3:** Patient treatment characteristics and outcomes.

		Duration (Days) Mean ± SD	
Treatment duration (*n* = 99)		13.2 ± 6.3	
Clinical improvement (*n* = 30)		3.4 ± 1.2	
Microbiological eradication (*n* = 35)		4.7 ± 2.1	
Relapse/reinfection (*n* = 5)		50.8 ± 27.5	
		***n***	**%**
Monotherapy		49	49.5
Combination therapy		50	50.5
Additional antimicrobial use *		70	70.7
	Caspofungin	29	29.3
	Colistin	26	26.3
	Teicoplanin	17	17.2
	Tigecycline	16	16.2
	Vancomycin	12	12.1
	Meropenem	10	10.1
	Levofloxacin	8	8.1
	Amikacin	7	7.1
	Linezolid	7	7.1
	Fosfomycin	5	5.1
	Liposomal Amphotericin-B	5	5.1
	Voriconazole	5	5.1
	Anidulafungin	3	3
	Trimethoprim-Sulfamethoxazole	3	3
	Gentamicin	2	2
	Ganciclovir	2	2
	Fluconazole	1	1
	Metronidazole	1	1
Ceftazidime–avibactam Side Effects		1 **	1

* More than one antimicrobial was used concurrently in some patients. ** Diarrhea was observed as a side effect.

**Table 4:** Patient mortality characteristics.

	*n* (%)	Mean ± SD
Mortality during CZA treatment (Days)	22 (17.2)	8.2 ± 5.8
	***n***	**%**
Successful treatment response	76	76.8
Mortality due to infection *	22	17.2
Mortality due to another infection episode	22	22.2
30-day mortality	48	48.5
90-day mortality **	72	72.7

* Mortality related to that infection episode in patients treated with CZA due to CRK growth. ** 30-day mortality is included in the 90-day mortality.

**Table 5:** Factors affecting post-therapy response.

	Treatment Success	Treatment Failure	*p*
Gender (M/F)	44/32	17/6	0.166
Age	63.1 ± 14.8	63.1 ± 18.24	0.709
Therapy (Combined therapy/Monotherapy)	37/39	13/10	0.510
Infection type			
Bloodstream infection	41	15	0.339
VAP	24	5	0.364
Intra-abdominal infection	5	1	1
Nosocomial pneumonia	4	0	0.570
SSI	4	0	0.570
Urinary tract infection	2	2	0.230
Decubitus infection	1	0	1
Duration of treatment	15.2 ± 5.1	6.9 ± 5.8	<0.001
Admission diagnoses			>0.05

SSI: Surgical site infection, M: Male, F: Female, VAP: Ventilator-associated pneumonia.

## Data Availability

The data are available upon reasonable request from the corresponding author.

## Abbreviations

The following abbreviations are used in this manuscript:

CRK	Carbapenem-resistant *Klebsiella* spp.
CZA	Ceftazidime–avibactam
ICU	Intensive care unit
MDR	Multidrug-resistant
ESBL	Extended-spectrum beta-lactamase
KPCs	*Klebsiella pneumoniae* carbapenemases
MBLs	Metallo-β-lactamases
OXA-48	Oxacillinase-48
CRE	Carbapenem-resistant *Enterobacteriaceae*
VAP	Ventilator-associated pneumonia
CDC	Centers for Disease Control and Prevention
CRP	C-reactive protein
MALDI-TOF MS	Matrix-assisted laser desorption/ionization time-of-flight mass spectrometry
MICs	Minimum inhibitory concentrations
EUCAST	European Committee on Antimicrobial Susceptibility Testing
SD	Standard deviation
SSI	Surgical site infection
CKD	Chronic kidney disease
COPD	Chronic obstructive pulmonary disease
Auto-HSCT	Autologous hematopoietic stem cell transplantation
SOT	Solid organ transplantation
CVE	Cerebrovascular event
IDSA	Infectious Diseases Society of America
DM	Diabetes mellitus
AIDS	Acquired immunodeficiency syndrome
BSI	Bloodstream infections
ALP	Alkaline phosphatase
GGT	Gamma-glutamyl transferase

## References

[1] Jorgensen S.C.J., Trinh T.D., Zasowski E.J., Lagnf A.M., Bhatia S., Melvin S.M., E Steed M., Simon S.P., Estrada S.J., Morrisette T. (2019). Real-World Experience With Ceftazidime-Avibactam for Multidrug-Resistant Gram-Negative Bacterial Infections. Open Forum Infect. Dis..

[2] Falcone M., Paterson D. (2016). Spotlight on ceftazidime/avibactam: A new option for MDR Gram-negative infections. J. Antimicrob. Chemother..

[3] Soriano A., Montravers P., Bassetti M., Klyasova G., Daikos G., Irani P., Stone G., Chambers R., Peeters P., Shah M. (2023). The Use and Effectiveness of Ceftazidime-Avibactam in Real-World Clinical Practice: EZTEAM Study. Infect. Dis. Ther..

[4] Yao Y., Zha Z., Li L., Tan H., Pi J., You C., Liu B. (2024). Healthcare-associated carbapenem-resistant Klebsiella pneumoniae infections are associated with higher mortality compared to carbapenem-susceptible K. pneumoniae infections in the intensive care unit: A retrospective cohort study. J. Hosp. Infect..

[5] Republic of Türkiye, Ministry of Health, General Directorate of Public Health (2025). National Healthcare-Associated Infections Surveillance Network: Antibiotic Resistance Report 2024.

[6] Shirley M. (2018). Ceftazidime-Avibactam: A Review in the Treatment of Serious Gram-Negative Bacterial Infections. Drugs.

[7] Tamma P.D., Heil E.L., Justo J.A., Mathers A.J., Satlin M.J., Bonomo R.A. (2024). Infectious Diseases Society of America 2024 Guidance on the Treatment of Antimicrobial-Resistant Gram-Negative Infections. Clin. Infect. Dis..

[8] Social Security Institution Presidency Communiqué on Amendment to the Social Security Institution Health Implementation Communiqué. *Official Gazette of the Republic of Turkey*, 28 April 2021; No. 31468. https://www.resmigazete.gov.tr/eskiler/2021/04/20210428M1-1.htm.

[9] Tumbarello M., Raffaelli F., Giannella M., Mantengoli E., Mularoni A., Venditti M., De Rosa F.G., Sarmati L., Bassetti M., Brindicci G. (2021). Ceftazidime-Avibactam Use for Klebsiella pneumoniae Carbapenemase-Producing K. pneumoniae Infections: A Retrospective Observational Multicenter Study. Clin. Infect. Dis..

[10] Calvo-Garcı’a A., Ibanez Zurriaga M.D., Ramı’rez Herra’iz E., Pe’rez Aba’nades M., Sa’ez Be’jar C., Morell Baladro’n A. (2022). Ceftazidime–avibactam: Effectiveness and safety in the clinical practice. A third hospital level experience. OFIL.

[11] Soriano A., Carmeli Y., Omrani A.S., Moore L.S.P., Tawadrous M., Irani P. (2021). Ceftazidime-Avibactam for the Treatment of Serious Gram-Negative Infections with Limited Treatment Options: A Systematic Literature Review. Infect. Dis. Ther..

[12] Yang T.-Y., Huang C.-T., Liu P.-Y., Lin Y.-T., Huang Y.-S., Chang P.-H., Tseng C.-H., Chang Y.-T., Lu P.-L., Chen Y.-C. (2025). Real-world use and treatment outcomes of ceftazidime-avibactam in gram-negative bacterial infection in Taiwan: A multicenter retrospective study. J. Infect. Public Health.

[13] Piroth L., Vitrat V., Le Moing V., Bret P., Brault Y., Greenwood W., Chopin M.-C., Vicaut E., Montravers P., Tattevin P. (2025). Real-world use, effectiveness, and safety of ceftazidime-avibactam: Results of the French cohort OZAVIE. Infect. Dis. Now.

[14] Balandín B., Ballesteros D., Pintado V., Soriano-Cuesta C., Cid-Tovar I., Sancho-González M., Pérez-Pedrero M.J., Chicot M., Asensio-Martín M.J., Silva J.A. (2022). Multicentre study of ceftazidime/avibactam for Gram-negative bacteria infections in critically ill patients. Int. J. Antimicrob. Agents.

[15] Yu J., Zuo W., Fan H., Wu J., Qiao L., Yang B., Li W., Yang Y., Zhang B. (2023). Ceftazidime-Avibactam for Carbapenem-Resistant Gram-Negative Bacteria Infections: A Real-World Experience in the ICU. Infect. Drug Resist..

[16] Todi S., Sathe P., Ramasubramanian V., Swaminathan S., Talwar D., Prayag P., Rao P.V., Sabnis K., Kamat S., Mane A. (2024). Real-World Evidence on Use of Ceftazidime-Avibactam in the Management of Gram-Negative Infections: A Retrospective Analysis. Cureus.

[17] Karaiskos I., Daikos G.L., Gkoufa A., Adamis G., Stefos A., Symbardi S., Chrysos G., Filiou E., Basoulis D., Mouloudi E. (2021). Ceftazidime/avibactam in the era of carbapenemase-producing Klebsiella pneumoniae: Experience from a national registry study. J. Antimicrob. Chemother..

[18] Onorato L., Di Caprio G., Signoriello S., Coppola N. (2019). Efficacy of ceftazidime/avibactam in monotherapy or combination therapy against carbapenem-resistant Gram-negative bacteria: A meta-analysis. Int. J. Antimicrob. Agents.

[19] Sobh G., Araj G.F., Finianos M., Sourenian T., Hrabak J., Papagiannitsis C.C., Chaar M.E., Bitar I. (2024). Molecular characterization of carbapenem and ceftazidime-avibactam-resistant Enterobacterales and horizontal spread of bla NDM-5 gene at a Lebanese medical center. Front. Cell Infect. Microbiol..

[20] Shields R.K., Horcajada J.P., Kamat S., Irani P.M., Tawadrous M., Welte T. (2024). Ceftazidime-avibactam in the treatment of patients with bacteremia or nosocomial pneumonia: A systematic review and meta-analysis. Infect. Dis. Ther..

[21] Horan T.C., Andrus M., Dudeck M.A. (2008). CDC/NHSN surveillance definition of health care-associated infection and criteria for specific types of infections in the acute care setting. Am. J. Infect. Control.

[22] European Committee on Antimicrobial Susceptibility Testing (EUCAST) (2022). Breakpoint Tables for Interpretation of MICs and Zone Diameters.

